# ‘Population mixing, socio-economic status and incidence of childhood acute lymphoblastic leukaemia in England and Wales – analysis by census ward’ and ‘Childhood leukaemia and population movements in France, 1990–2003’

**DOI:** 10.1038/sj.bjc.6604551

**Published:** 2008-09-30

**Authors:** R Wakeford

**Affiliations:** 1 The Dalton Nuclear Institute, The University of Manchester, Pariser Building – G Floor, PO Box 88, Sackville Street, Manchester, M60 1QD, UK

**Sir,**

The recent publications of Stiller *et al* (2008)and Bellec *et al* (2008) make valuable contributions to the understanding of how population movements and mixing influence the risk of childhood leukaemia (CL) and provide further evidence of a major role for infection in its aetiology. The authors refer to the earlier work of Kinlen and his colleagues (e.g. Kinlen *et al*, 1995; Kinlen, 2006) who investigated the effect of marked influxes of people into rural areas (with consequent increases in population) upon the risk of CL in these areas. However, the results presented in the recent publications do not allow certain relevant comparisons to be made with the earlier work, and it would add greatly to the interest of the studies if a few additional details could be provided.

Stiller *et al* (2008) found that for young children aged 1–4 years, the incidence of childhood acute lymphoblastic leukaemia in the census wards of England and Wales during 1986–1995 was raised in wards where in-migrants (those resident in the ward at the 1991 census, but elsewhere 1 year previously) came from a greater diversity of originating wards. Bellec *et al* (2008) considered the incidence of CL in France during 1990–2003 in relation to population movements between communes during the inter-census period 1990–1999 and found that, particularly for the 0–4 year age group, incidence in isolated communes was higher when the proportion of in-migrants from distant areas was greatest. A material population increase requires a high level of in-migration, but the reverse is not necessarily the case, and I wonder if the wards and isolated communes showing raised rates of CL in these two recent publications experienced marked population increases? I appreciate that the study of Stiller *et al* (2008) involved only a single census, but it may not be difficult to determine, even with relatively crude population data, whether the wards with CL excesses showed any concentration in areas known to have experienced population increases since the previous census.

On a further point, Stiller *et al* (2008) state that the association between CL and diversity of incomers, ‘especially in rural areas’, is consistent with the findings of Kinlen and colleagues, but separate results for such rural areas are not presented. Would it be possible for results to be provided for rural wards with the greatest diversity of incomers?
